# Verification in an animal study of the appropriate settings for a novel radiofrequency generator in radiofrequency ablation therapy for residual intraductal lesions after endoscopic papillectomy (with video)

**DOI:** 10.1111/den.14986

**Published:** 2025-01-20

**Authors:** Kenjiro Yamamoto, Takao Itoi, Atsushi Sofuni, Takayoshi Tsuchiya, Reina Tanaka, Ryosuke Tonozuka, Kazumasa Nagai, Yukitoshi Matsunami, Hiroyuki Kojima, Hirohito Minami, Noriyuki Hirakawa, Kyoko Asano, Shuntaro Mukai

**Affiliations:** ^1^ Department of Gastroenterology and Hepatology Tokyo Medical University Tokyo Japan

**Keywords:** ampullary adenoma, animal experiment, endoscopic papillectomy, radiofrequency ablation, residual intraductal lesion

## Abstract

Endoscopic intraductal radiofrequency ablation (ID‐RFA) can curatively treat residual intraductal lesions after endoscopic papillectomy. This study aimed to verify the tissue invasiveness of ID‐RFA using a novel RF generator and to explore its appropriate settings in an animal experiment, followed by a small clinical study. Pig liver specimens were ablated using a dedicated RF catheter and two RF generators to investigate structural differences between them and the ablation effects produced under various voltage and power settings. Appropriate settings for the novel generator were sought to provide an ablation effect equivalent to that with the recommended settings for a conventional generator. The ablation effect was also observed at various ablation times in vitro. Then we performed ID‐RFA in five patients. Each generator has a different structure, and no novel generator settings are identical to the recommended conventional generator settings. Obtaining adequate ablation requires both sufficient power and sufficient voltage. Based on the validation experiments, we concluded that the appropriate novel generator settings were 125 Vp and 30 W for 30 s. In the clinical study, good tumor ablation was obtained with no recurrence after a single ID‐RFA treatment, although the incidence of ductal stricture was relatively high. ID‐RFA for residual intraductal lesions may potentially be curative. However, excessive ablation should be avoided. To ensure safe and effective ID‐RFA, a thorough understanding of the RF generator specifications is required.

## INTRODUCTION

Endoscopic papillectomy (EP) is a treatment for neoplasms arising in the duodenal papilla and is the only curative endoscopic treatment for biliopancreatic neoplasms. Several guidelines recommend EP for ampullary adenomas that do not extend into the pancreatic bile ducts and can be resected en‐bloc.^1^, ^2^, ^3^ Recently, evidence has been reported regarding expanding the indications for EP to adenomas with lateral extension that are difficult to resect en‐bloc and to carcinomas with a low risk of lymph node metastasis.^4^, ^5^, ^6^, ^7^, ^8^ Furthermore, endoscopic intraductal radiofrequency ablation (ID‐RFA) can now achieve radical treatment of residual neoplastic tissue extending from the papilla into the common bile duct (CBD) and/or the pancreatic duct after EP by inducing coagulative necrosis of the target tissue.^9^, ^10^, ^11^, ^12^, ^13^

Our unit previously reported the effects of the RFA catheter in an ex vivo model under various powers and times.^14^ However, a novel RF generator has recently been introduced. Our previous study suggested that this novel generator may have a different tissue invasiveness compared with the conventional generator.^15^ Therefore, the effectiveness of ID‐RFA using this new generator needs to be reassessed. Thus, the present study examined the tissue invasiveness of ID‐RFA using this novel generator under various conditions and explored the settings suitable for ID‐RFA of intraductal residual lesions after EP in ampullary adenoma.

## PROCEDURE

### RFA devices

**Figure 1:**
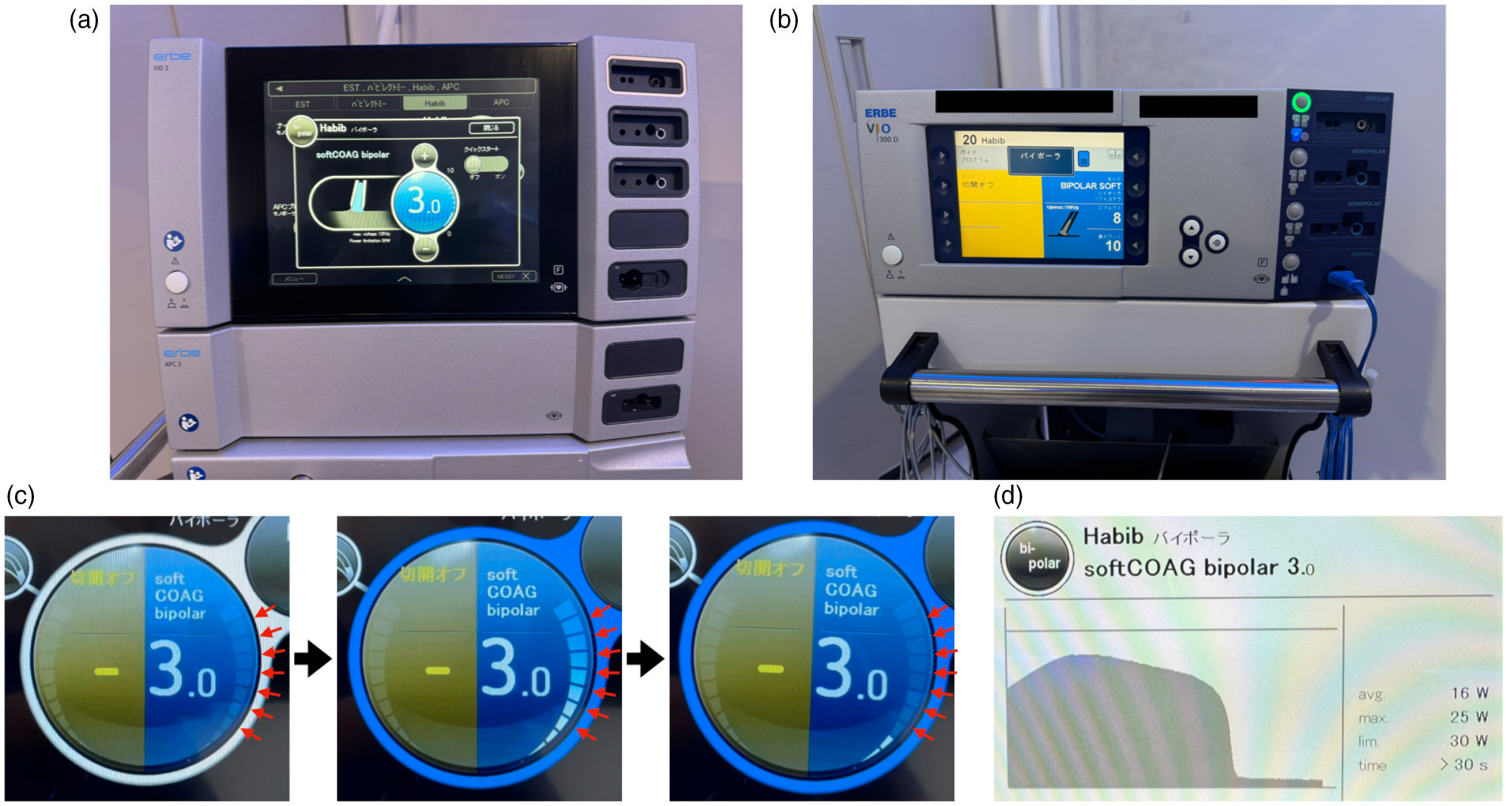
Radiofrequency (RF) generator systems. Two RF generator systems were used in this study: (a) VIO3 and (b) VIO300D (Erbe, Tübingen, Germany). (c) In the VIO3, real‐time output power levels during energization are displayed on the screen. (d) In addition, the average output, maximum output, and output graph are displayed on the screen after output. In contrast, only the average output and maximum output are displayed on the screen in the VIO300D.

An RFA catheter (Habib EndoHPB; Boston Scientific, Marlborough, MA, USA) and two RF generator systems (VIO3 and VIO300D; Erbe, Tübingen, Germany) were used. Device characteristics are shown in Figures 1 and S1 and Table S1.

### Animal subjects

For the in vitro experiment, resected fresh livers from an adult Yorkshire pig were used. The organ was removed within 30 min after the pig was killed and then processed and transported on ice to our institute. The experiment was started within 8 h postmortem and was performed at room temperature. In the in vivo experiment, healthy and living Yorkshire pigs were used. All pigs were provided water alone for 24 h before the endoscopic procedures and fasted overnight beforehand. Intravenous ketamine (0.2 mg/kg) and 0.2% xylazine (0.1 mg/kg, Selactar; Bayer Yakuhin, Tokyo, Japan) were used to induce general anesthesia, which was maintained by using 2–5% isoflurane. Atropine (1 mg) was administered to reduce secretions.

### In vitro experiment

#### Verification of the ablation effects of 10 W for VIO3 and VIO300D (recommended settings)

The RFA settings of VIO3 were bipolar 1.0, with voltage of 50 Vp and power of 10 W. In contrast, the RFA settings of VIO300D were voltage of Effect 8 (190 Vp) and power of 10 W (recommended settings). Each RF application was performed for 90 s.

#### Verification of the ablation effect of each voltage at 10 W for VIO300D

Radiofrequency ablation application using VIO300D was performed at 10 W for 90 s in eight voltage patterns: step‐by‐step at Effect 1 (55 Vp), 2 (70 Vp), 3 (90 Vp), 4 (110 Vp), 5 (135 Vp), 6 (155 Vp), 7 (175 Vp), and 8 (190 Vp).

#### Verification of VIO3 output settings expected to have the same ablation effect as the recommended settings of VIO300D

The RF application using VIO3 was performed step‐by‐step at powers of 10, 20, 30, 40, and 50 W for 15, 30, 60, 90, and 120 s, respectively. As a control, RF application using VIO300D with the recommended settings was performed for the same times. The same application was repeated four times.

All in vitro experiments were performed as follows. An RFA catheter was inserted into the resected fresh liver over the guidewire and connected to a generator. Immediately after each application, the liver was cut along the guidewire using a pathology knife in order to evaluate the ablation area. The ablation area was macroscopically measured with a scale as the maximum lengths of the short axis and long axis (Fig. S2). Sufficient ablation was defined as the detection of a yellowish white change around and between each electrode. Insufficient ablation was defined as the detection of yellowish white change only around each electrode. Average and maximum outputs were recorded for the two RF generator systems. Also, the time until the output dropped and the output graph were recorded in VIO3.

### In vivo experiment

#### Observation of coagulation changes over time with different ablation times at bipolar 3.0 (125 Vp, 30 W) for VIO3 ID‐RFA

##### Endopancreatic ID‐RFA

Under general anesthesia, all pigs underwent endopancreatic ID‐RFA. A guidewire was placed in the pancreatic duct. The RFA catheter was inserted so that the second ring from the tip was located at the pancreatic duct orifice. The RFA catheter was connected to the VIO3 generator and RF power was applied for a set time. The RF application was performed step‐by‐step at bipolar 3.0 (125 Vp, 30 W) for 30, 60, 90, and 120 s. After ablation, a plastic stent was placed to prevent adverse events.

##### Endoscopic, macroscopic, and microscopic assessments after intervention

Immediately and 1 week after each application, coagulation changes were endoscopically observed. All pigs were then killed to macroscopically assess the coagulation changes. The resected specimens were fixed in formalin and embedded in paraffin for hematoxylin and eosin staining and sectioning. The serial sections of whole resected specimens were examined at 5 mm intervals, and the area of necrosis was evaluated microscopically in each specimen.

#### Ethical approval for animal experiments

All animal experiments were approved by the Institutional Animal Ethics Committee (approval number NRT220807).

### Preliminary clinical study

Guided by the results of the animal experiments, five consecutive patients underwent VIO3 ID‐RFA for residual intraductal adenoma after EP from March 2020 to July 2021 at Tokyo Medical University Hospital. The inclusion criterion was the presence of intraductal adenoma confirmed by intraductal biopsy at the end of the common bile duct (CBD). This clinical study was approved by the Institutional Review Board of Tokyo Medical University (approval number T2023‐0053) and performed in accordance with the relevant guidelines and regulations, including the Declaration of Helsinki. Informed consent for the ID‐RFA procedure was obtained from all participants. This is a retrospective observational study that does not contain identifiable personal information. We published details about the study on our website to allow patients to choose to opt out if they wished.

#### ID‐RFA procedure

The ID‐RFA procedures were performed with the patient under general anesthesia. Endoscopic retrograde cholangiopancreatography was performed using a large‐channel duodenoscope (TJF‐260/TJF‐Q290V; Olympus, Tokyo, Japan) by experienced pancreatobiliary endoscopists (K.Y., T.I.). Cholangiography and intraductal ultrasonography confirmed the biliary stricture as well as its length, diameter, and position at the end of the CBD. The RFA catheter was inserted across the end of the CBD under fluoroscopic guidance, and the RF generator VIO3 was applied at bipolar 3.0 (125 Vp, 30 W) for 30 s under pancreatic stent placement. After ablation, biliary stent placement was performed with a fully covered self‐expandable metal stent for perforation and papillary stricture. The procedure has previously been detailed.^16^

#### Follow‐up

Biliary and pancreatic stents were removed by follow‐up endoscopy 1–2 months after RFA. Subsequent follow‐up endoscopies were performed every 3–6 months within the first year after RFA and every 6 months to 1 year thereafter. At all follow‐up endoscopies, biopsy was performed at the margin of the RFA site to assess adenoma recurrence. If the biopsy was positive for adenomatous tissue, additional RFA treatment was planned.

#### Outcome measures

The primary end‐point was ID‐RFA clinical success, defined as histological absence of recurrence based on extensive follow‐up biopsies from the papillectomy site and distal bile duct. The secondary end‐point was procedure‐related adverse events. Sex, age, pathological characteristics, number of ID‐RFA procedures, and the need for complementary interventions were recorded.

## RESULTS

### Verification of the ablation effects of 10 W for VIO3 and 10 W for VIO300D (recommended setting)

The macroscopic ablation areas are shown in Figure S3. The ablation effect was insufficient with VIO3 but sufficient with VIO300D. The maximum power outputs were 5.4 W in VIO3 and 9 W in VIO300D.

### Verification of the ablation effect of each voltage and 10 W for VIO300D

The macroscopic ablation areas and maximum power output are shown in Figure S4. At voltages above 90 Vp in Effect 3, the maximum power output was close to the set power and, as a result, sufficient ablation areas were observed.

### Verification of VIO3 output settings expected to have the same ablation effect as the recommended settings of VIO300D

**Figure 2:**
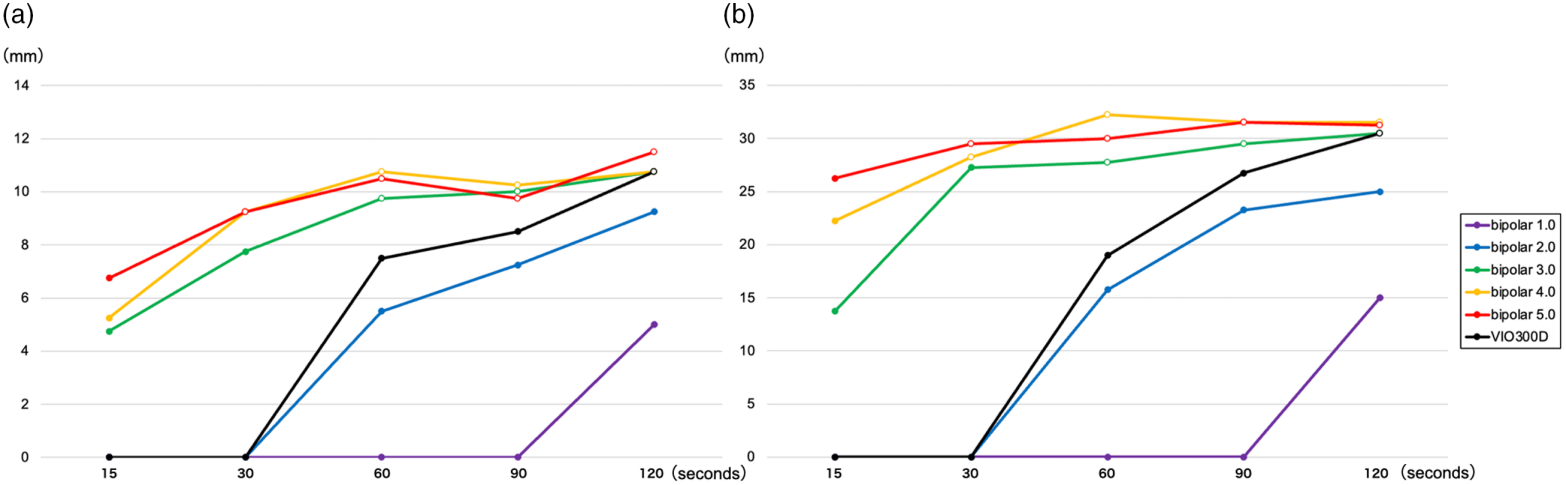
Ablation area of various output settings of VIO3 and the recommended settings of VIO300D (both Erbe, Tübingen, Germany). (a) The mean lengths of the short axis at bipolar 1.0 (50 Vp, 10 W) and at 15, 30, 60, 90, and 120 s were 0, 0, 0, 0, and 5 ± 0 mm, respectively. The mean lengths of the short axis at bipolar 2.0 (70 Vp, 20 W) and at 15, 30, 45, 60, 90, and 120 s were 0, 0, 5.5 ± 0.6, 7.3 ± 0.5, and 9.3 ± 0.5 mm, respectively. The mean lengths of the short axis at bipolar 3.0 (125 Vp, 30 W) and at 15, 30, 60, 90, and 120 s were 4.8 ± 0.5, 7.8 ± 0.5, 9.8 ± 0.5, 10 ± 0, and 10.8 ± 0.5 mm, respectively. The mean lengths of the short axis at bipolar 4.0 (150 Vp, 40 W) and at 15, 30, 60, 90, and 120 s were 5.3 ± 0.5, 9.3 ± 0.5, 10.8 ± 0.5, 10.3 ± 0.5, and 10.8 ± 0.5 mm, respectively. The mean lengths of the short axis at bipolar 5.0 (175 Vp, 50 W) and at 15, 30, 60, 90, and 120 s were 6.8 ± 1.0, 9.3 ± 1.0, 10.5 ± 0.6, 9.8 ± 0.5, and 11.5 ± 0.6 mm, respectively. The mean lengths of the short axis for VIO300D (190 Vp, 10 W) and at 15, 30, 60, 90, and 120 s were 0, 0, 7.5 ± 0.6, 8.5 ± 0.6, and 10.8 ± 0.5 mm, respectively. (b) The mean lengths of the long axis at bipolar 1.0 (50 Vp, 10 W) and at 15, 30, 60, 90, and 120 s were 0, 0, 0, 0, and 15 ± 0 mm, respectively. The mean lengths of the long axis at bipolar 2.0 (70 Vp, 20 W) and at 15, 30, 60, 90, and 120 s were 0, 0, 15.8 ± 1.0, 23.3 ± 1.3, and 25 ± 0.8 mm, respectively. The mean lengths of the long axis at bipolar 3.0 (125 Vp, 30 W) and at 15, 30, 60, 90, and 120 s were 13.8 ± 1.0, 27.3 ± 1.0, 27.8 ± 1.3, 29.5 ± 1, and 30.5 ± 0.6 mm, respectively. The mean lengths of the long axis at bipolar 4.0 (150 Vp, 40 W) and at 15, 30, 60, 90, and 120 s were 22.3 ± 2.1, 28.3 ± 4.2, 32.3 ± 3.6, 31.5 ± 1.7, and 31.5 ± 0.6 mm, respectively. The mean lengths of the long axis at bipolar 5.0 (175 Vp, 50 W) and at 15, 30, 60, 90, and 120 s were 26.3 ± 1.3, 29.5 ± 1.9, 30 ± 0, 31.5 ± 1.3, and 31.3 ± 1.3 mm, respectively. The mean lengths of the long axis for VIO300D (190 Vp, 10 W) and at 15, 30, 60, 90, and 120 s were 0, 0, 19 ± 1.2, 26.8 ± 1.0, and 30.5 ± 0.6 mm, respectively. In the figure, an open circle indicates that the output is down, and a closed circle indicates that the output is not down.

**Figure 3:**
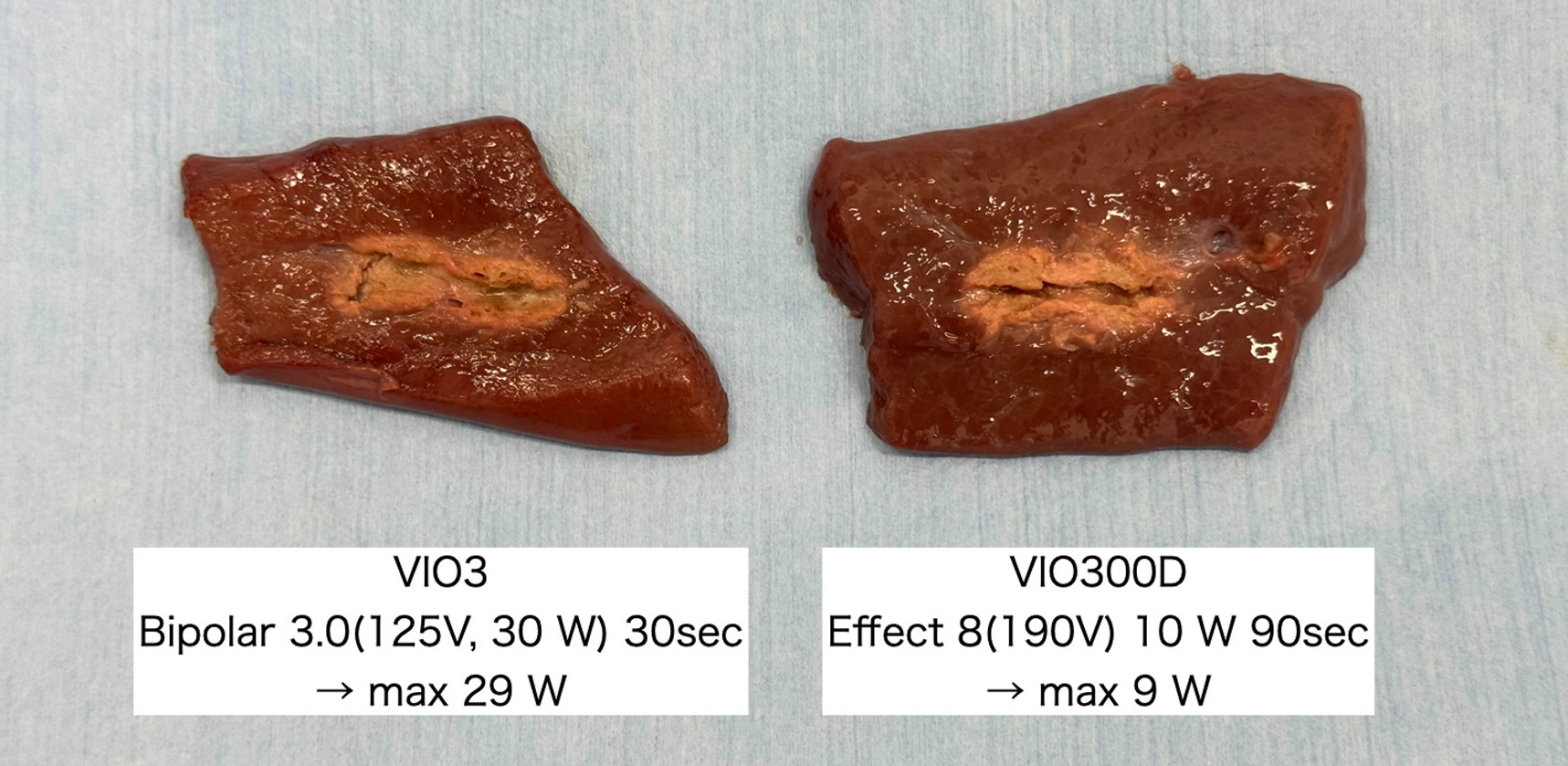
Ablation effect of VIO3 (Erbe, Tübingen, Germany) at bipolar 3.0 (125 Vp, 30 W) for 30 s. The ablation effect at bipolar 3.0 (125 Vp, 30 W) for 30 s was equivalent to that obtained with the recommended VIO300D (Erbe) settings. The maximum power was 29 W in VIO3 and 9 W in VIO300D.

The ablation area outcomes are shown in Figure 2. The VIO3 settings, which have the same linear pattern as that of VIO300D, were not observed in this validation experiment. The most similar settings were bipolar 3.0 (125 Vp, 30 W) for 30 s, followed by bipolar 2.0 (125 Vp, 30 W) for 120 s. The ablation effects at bipolar 3.0 (125 Vp, 30 W) are shown in Figures 3 and S5.

The average output, maximum output, and the time until the output dropped are shown in Tables S2–S4. When ablation was performed at higher output settings, the output power declined before the specified energization time.

### Observation of coagulation changes with different ablation times at bipolar 3.0 (125 Vp, 30 W) for VIO3 ID‐RFA

**Figure 4:**
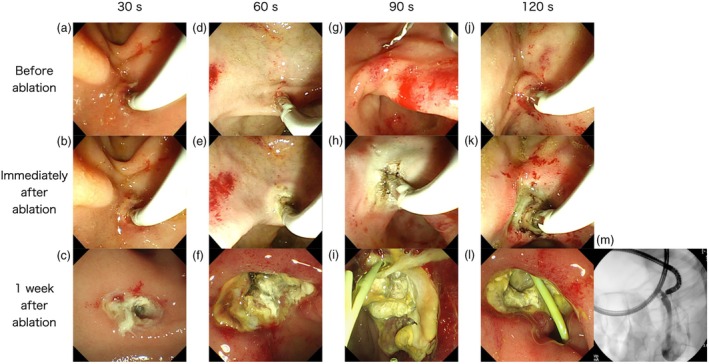
Change in the ablation area over time based on coagulation time with 30 W. (a–c) (30 s) Immediately after ablation, the yellowish white area was 6 × 6 mm in size. One week after ablation, the necrotic area was 8 × 8 mm in size. (d–f) (60 s) Immediately after ablation, the yellowish white area was 10 × 9 mm in size. One week after ablation, the necrotic area was 13 × 10 mm in size. (g–i) (90 s) Immediately after ablation, the yellowish white area was 25 × 15 mm in size. One week after ablation, the necrotic area was 30 × 17 mm in size. (j–m) (120 s) Immediately after ablation, the yellowish white area was 22 × 20 mm in size. One week after ablation, the necrotic area was 26 × 24 mm in size. In addition, necrotic changes formed fistulas connecting to the colon.

Figure 4 shows endoscopic images of the pancreatic orifice immediately and 1 week after ablation. Immediately after ablation, a yellowish white area around the pancreatic orifice spread in proportion to the ablation time. One week after ablation, a wider area became ulcerated than the observed yellowish white area. In particular, with “30 W, 120 s,” necrotic changes formed fistulas connecting to the colon.

Microscopically, the necrotic area matched the ablation area in all specimens (Fig. S6). There was no obvious histological evidence of acute pancreatitis in the serial sections of whole resected specimens.

### Preliminary clinical study

Five male patients underwent ID‐RFA treatment for intraductal extension of ampullary adenoma; one case was included in a previous publication.^16^ The ID‐RFA treatment outcomes are shown in Tables 1 and S5. All patients underwent one RFA session. Prophylactic CBD and pancreatic duct stents were placed in all RFA sessions. All patients underwent endoscopic surveillance after the completion of treatment over a median follow‐up of 24 months (range 12–48) with intraductal biopsy specimens showing no intraductal neoplasm.

**Table 1:** Summarized data on clinical outcomes of five patients with intraductal extension of ampullary neoplasms treated with VIO3 intraductal radiofrequency ablation (ID‐RFA)

Case, *n*	5
Age, years, median (range)	51 (37–82)
Male, *n* (%)	5 (100)
Tumor size, mm, median (range)	16 (15–22)
EP pathology, *n* (%)	
High‐grade adenoma	4 (80)
Carcinoma in adenoma	1 (20)
Intraductal extension, mm, median (range)	10 (8–15)
Intraductal pathology, *n* (%)	
Low‐grade adenoma	3 (60)
High‐grade adenoma	2 (40)
Complementary interventions before ID‐RFA, *n*	
Algon plasma coagulation	5
Snare resection	1
ID‐RFA session, *n*	1
Adverse events, *n* (%)	B‐duct stricture, 2 (40)
Follow‐up period, months, median (range)	24 (12–48)
Recurrence, *n*	0

B‐duct, bile duct; EP, endoscopic papillectomy.

As adverse events, two patients had bile duct strictures; one occurred 2 months after RFA and the other occurred 3 months after RFA with symptoms. These strictures were treated with multiple plastic stents for 4 months in one patient and fully covered metal stents for 3 months in one patient.

## DISCUSSION

Through in vitro and in vivo experiments, we identified the differences in performance between the RFA device according to the output settings. We also confirmed the mechanical characteristics provided by two factors – voltage (Vp) and power (W) – regarding the ablation effect of ID‐RFA. First, each RF generator has different output settings. As shown in Table S1, the same output settings as those recommended for VIO300D cannot be selected for VIO3. Second, power is the “power limitation” and is controlled by the RF generator so that it does not exceed the set power. Therefore, even if the power is set to 10 W, the power generated is small when the voltage is low, and thus a sufficient ablation effect cannot be obtained (Fig. S3). Therefore, a certain amount of voltage is required to produce a set amount of power.

Based on the understanding of these mechanisms of RF generators, we found that the VIO3 settings that produce a similar ablation effect as the recommended settings of VIO300D are bipolar 3.0 (125 Vp, 30 W) for 30 s (Fig. 3). We further verified changes in tissue degeneration over time after RFA and found that the yellowish white tissue detected immediately after RFA widely degenerated and necrosed with time (Fig. 4). In other words, tissue degeneration occurs over a wider range than that observed by the naked eye.

Some things should be kept in mind when performing RFA with VIO‐ESU. As shown in Tables S2–S4, VIO‐ESU has a function in which the generator automatically reduces the output power when sufficient ablation is obtained (i.e. when tissue resistance becomes high), even in the process of energizing time. Thermography shows that when the probe is energized for 90 s, the output drops 30 s after the start of energization, but the probe continues to be heated thereafter (Fig. S7). As a result, the longer the energization time, the greater the cauterization effect (Fig. 4). Then the ablation area spreads slowly after the output power drops (Fig. 2). Even with low thermal energy, prolonged energization will cause coagulation degeneration to the extent that perforation occurs. Therefore, energization should be terminated when the output falls. The VIO3, which allows the output to be monitored, is a safer ESU for RFA compared with the VIO300D.

Based on the above validation results, we conducted a clinical study using suitable settings for VIO3 ID‐RFA (Tables 1 and S5). All patients had good tumor ablation results, with no recurrence after a single RFA treatment during the observation period. However, as in previous reports,^12^, ^13^ a relatively high incidence of ductal stricture was observed. Power settings of 125 Vp and 30 W for 30 s for VIO3‐ID‐RFA may provide a good tumor ablation effect in clinical practice, but may be somewhat excessive from the viewpoint of strictures.

Figure S8 shows the current flows of an RFA catheter. In general, the ablation effect spreads from the periphery of the ring to the dumbbell and then oval shapes because the current flows in a circular pattern from the central side of the two electrodes. However, when the second electrode is positioned at the papillary orifice, the ablation effect might spread, as shown in Figure S9. A greater ablation effect around the second electrode can be obtained in the early phase of the current application time when the area around the first electrode is less cauterized.

Accordingly, we provide such tips in Figure S10 and Video S1. For safe and effective RFA treatment, the target lesion should be ablated with the second electrode (ring). An advantage of ID‐RFA of the papillary region is the real‐time observation of ablation changes, and the current flow is terminated when a white color change is confirmed well beyond the expected range. We do not necessarily need to ablate intraductal lesions confined to the epithelium at the recommended time because an area slightly wider than the yellowish white degeneration area will disappear over time.

The manufacturer has recently announced the recommended settings for VIO3: bipolar 1.4–2.0, 90 s. These settings slightly suppress the ablation effect compared with the optimal settings of this study in Figure 2. However, in actual clinical practice, considering factors such as respiratory fluctuations, keeping the electrode in the appropriate position for a short current application time is not easy. Therefore, optimal settings with a 90 s current application time may be desirable, as with the optimal settings for the VIO300D.

This study has some limitations. First, sample sizes were small in the experimental animal and clinical studies. Second, experimental studies were conducted using normal porcine liver and pancreatic duct. Impedance may differ between normal swine tissue and human cancer tissue. This swine tissue may not necessarily replicate the pathophysiological substrates of the human bile and pancreatic duct tissues analyzed in the clinical studies. However, we believe that these studies provide insight into the basic understanding of RFA and will be valuable, not only for post‐EP residual lesions, but also for RFA treatment in general.

In conclusion, RFA for residual intraductal lesions after EP may be a potentially curative and useful treatment, although post‐RFA ductal strictures are a concern. The RF generator requires a thorough understanding of its specifications, because the settings vary among devices. To ensure safe and effective RFA treatment, it is also important to understand how ablation spreads from the RFA catheter and changes over time. These experiments' findings indicate that further clinical data are needed.

## CONFLICT OF INTEREST

Authors T.I. and T.T. are consultants for Olympus, Boston Scientific Japan, and Gadelius Medical K.K. The other authors declare no conflict of interest for this article.

## FUNDING INFORMATION

None.

## ETHICS STATEMENT

Approval of the research protocol by an Institutional Reviewer Board: This clinical study was approved by the Institutional Review Board of Tokyo Medical University (approval number T2023‐0053) and was conducted in accordance with the relevant guidelines and regulations (Declaration of Helsinki).

Informed Consent: This retrospective observational study does not contain identifiable personal information. We published details about the study on our website to allow patients to opt out if they wish.

Registry and the Registration No. of the study/trial: N/A.

Animal studies: All animal experiments were approved by the Institutional Animal Ethics Committee (approval number NRT220807).

## Supporting information


**Figure S1** Radiofrequency ablation catheter.
**Figure S2** Practical in vitro experiment.
**Figure S3** Ablation effect of 10 W for VIO3 and 10 W for VIO300D (recommended setting).
**Figure S4** Ablation effects of each voltage with 10 W for VIO300D.
**Figure S5** Ablation effects of each time for VIO3 at bipolar 3.0 (125 Vp, 30 W).
**Figure S6** Resected specimen with an ablation time of 60 s stained with hematoxylin and eosin.
**Figure S7** Observation of temperature change of electrode by thermography.
**Figure S8** Schema of the spread of the ablation effect during radiofrequency ablation.
**Figure S9** Ablation effect produced by differences in current density.
**Figure S10** The demonstration of tips on effective ablation in intraductal radiofrequency ablation using VIO300D with the recommended setting.
**Table S1** Characteristics of the bipolar settings in each radiofrequency generator.
**Table S2** Data of average output for each setting.
**Table S3** Data of maximum output for each setting.
**Table S4** Time until output down in each setting.
**Table S5** Detailed data on clinical outcomes of five patients with intraductal extension of ampullary neoplasms treated with VIO3 intraductal radiofrequency ablation.

**Video S1** The second ring was firmly positioned over the lesion and energized for 20 s.
