# Supplementary material for: Verification in an animal study of the appropriate settings for a novel radiofrequency generator in radiofrequency ablation therapy for residual intraductal lesions after endoscopic papillectomy (with video)
Source: Dig Endosc. 2025 Jan 20;37(6):704–11. doi: 10.1111/den.14986 (PMC12162417; doi:10.1111/den.14986)
Supplement: Supplementary file 1 — Figure S1 Radiofrequency ablation catheter. Figure S2 Practical in vitro experiment. Figure S3 Ablation effect of 10 W for VIO3 and 10 W for VIO300D (recommended setting). Figure S4 Ablation effects of each voltage with 10 W for VIO300D. Figure S5 Ablation effects of each time for VIO3 at bipolar 3.0 (125 Vp, 30 W). Figure S6 Resected specimen with an ablation time of 60 s stained with hematoxylin and eosin. Figure S7 Observation of temperature change of electrode by thermography. Figure S8 Schema of the spread of the ablation effect during radiofrequency ablation. Figure S9 Ablation effect produced by differences in current density. Figure S10 The demonstration of tips on effective ablation in intraductal radiofrequency ablation using VIO300D with the recommended setting. Table S1 Characteristics of the bipolar settings in each radiofrequency generator. Table S2 Data of average output for each setting. Table S3 Data of maximum output for each setting. Table S4 Time until output down in each setting. Table S5 Detailed data on clinical outcomes of five patients with intraductal extension of ampullary neoplasms treated with VIO3 intraductal radiofrequency ablation. [file DEN-37-704-s002.zip › den14986-sup-0001-Data S1.docx]

Supporting information

**Fig. S1 RFA catheter**

This RFA catheter is a bipolar 8-Fr (2.6-mm) probe that is 1.8-m long, compatible with standard side-viewing endoscopes, and passes over 0.035-inch guidewires. The catheter has two ring electrodes 8 mm apart, with the distal electrode 5 mm from the leading edge, and provides local coagulative necrosis over a 2.5-cm length.

**Fig. S2 Practical in vitro experiment**

a. An RFA catheter was inserted into the resected fresh liver over the guidewire and connected to a generator.

b. To determine the ablation area, the maximum lengths of the short axis (i.e., the penetration) and long axis (i.e., the spreading) were macroscopically measured with a scale.

**Fig. S3 Ablation effect of 10 W for VIO3 and 10 W for VIO300D (the recommended setting)**

a. The ablation effect was insufficient for VIO3 while sufficient ablation effect was obtained for VIO300D.

b. The maximum power outputs were 5.4 W in VIO3 and 9 W in VIO300D.

**Fig. S4 Ablation effects of each voltage with 10 W for VIO300D**

The maximum power was 5 W at 55 Vp in Effect 1 and 7 W at 70 Vp in Effect 2. At voltages above 90 Vp in Effect 3, the maximum power output was 9 W.

**Fig. S5 Ablation effects of each time for VIO3 at bipolar 3.0 (125 Vp, 30 W)**

The maximum power output was 24 W for 15 s. At an ablation time of more than 30 s, the maximum power output was close to the set power, and sufficient ablation areas were observed.

**Fig. S6 Resected specimen with an ablation time of 60 s stained with hematoxylin and eosin**

a. Low-power image showing degeneration in the center of the specimens.

b. In the red area, a high-power image shows necrotic tissue in the center of the specimens.

c. In the blue area, a high-power image shows granulation tissue comprising fibroblasts and blood vessels, surrounded by regenerating pancreatic ducts.

d. In the blue area, a high-power field shows normal pancreatic acinar cells.

**Fig. S7** **Observation of temperature change of electrode by thermography**

Electrodes were attached to resected fresh livers, and the difference in temperature change of the electrodes during the 30-s (a–e) and 90-s (f–j) periods of electrical current was verified using a thermograph.

a) The output was stopped at 30 s of energization time (red circle). The average power was 14 W and the maximum was 17 W.

b-d) At 30 s, the electrode was 92.7°C. Then, at 60 s, it was 47.1°C, and at 90 s, it was 37.7°C. The temperature of the electrode decreased when the output was stopped.

e) Macroscopic finding showing the ablation area at the 30-s period of electrical current.

f) The output decreased at 25 se of energization time, but the low output was maintained thereafter (red circle). The average was 5.1 W, and the maximum was 18 W.

g-i)The electrode was 86.7°C at 30 s, , 59.2°C at 60 s, 61.7°C at 90 s. The electrode emits energy even at low power.

j) Macroscopic finding showing the ablation area at the 90-s period of electrical current. The ablation area is wider than that at the 30-s period of electrical current (Fig. S7e).

**Fig. S8 Schema of the spread of the ablation effect during RFA**

Because the current flows in a circular pattern from the central side of the two electrodes (a), the ablation effect spreads from the periphery of the ring (b) to the dumbbell (c) and then oval (d) shapes.

**Fig. S9 Ablation effect produced by differences in current density**

a. In the bile duct orifice, where there is not enough tissue around the second electrode, the area around the second electrode is strongly ablated because the current density varies depending on the area of the electrodes in contact.

b. The ablation effect is spread around the central electrode, so that if, for example, only half of the electrodes are in contact, a greater ablation effect can be obtained in the early phase of the procedure.

**Fig. S10 The demonstration of tips on effective ablation in ID-RFA using VIO300D with the recommended setting.**

a. After endoscopic papillectomy for ampullary adenoma, a residual adenoma lesion on the duodenal wall was detected histologically.

b. Cholangiography revealed residual intraductal infiltration 10 mm in diameter at the end of common bile duct.

c. The second ring was firmly positioned over the lesion and energized for 20 s. No apparent change in color tone was seen in the endoscopic image because ablation occurs from the center of the ring.

d. The catheter was withdrawn slightly and energized for another 20 s. A change in color tone can be seen.

e. The tip of the second ring is then positioned over the orifice and energized for 20 s. The tissue around the tip of the ring is strongly ablation.

f. A sufficient ablation effect can be confirmed even with a shorter energization time than recommended.

**Video legend**

The second ring was firmly positioned over the lesion and energized for 20 s.

An apparent change in color tone was not seen in the endoscopic image because ablation occurs from the center of the ring.

The catheter was withdrawn slightly and energized for another 20 s.

A change in color tone can be seen.

The tip of the second ring is then positioned over the orifice and energized for 20 s.

The tissue around the tip of the ring is strongly ablated.

Table S1. Characteristics of the bipolar settings in each RF generator

| VIO3 | | |  | VIO300D | | |
| --- | --- | --- | --- | --- | --- | --- |
| Bipolar | Voltage (Vp) setting  Max. voltage | Power (W) setting  Power limitation |  | Effect | Voltage (Vp) setting  Max. voltage | Power (W) setting  Power limitation |
| 1.0 | 50 | 10 |  | 1 | 55 | 1–120  arbitrarily selectable |
| 2.0 | 70 | 20 |  | 2 | 70 |  |
| 3.0 | 125 | 30 |  | 3 | 90 |  |
| 4.0 | 150 | 40 |  | 4 | 110 |  |
| 5.0 | 175 | 50 |  | 5 | 135 |  |
| 6.0 | 175 | 60 |  | 6 | 155 |  |
| 7.0 | 200 | 70 |  | 7 | 175 |  |
| 8.0 | 200 | 80 |  | 8 | 190 |  |

A characteristic of VIO3 is that the voltage (Vp) and power (W) are pre-set. In “bipolar” notation, they are set in 100 steps from 0.1 to 10. The figure shows the bipolar setting of VIO3 corresponding to the Effect setting of VIO300D. A characteristic of the bipolar setting of VIO300D is that the operator can arbitrarily select the Effect, which is the voltage (Vp) setting, and the Power (W) setting. Specifically, the Effect can be selected from 55 to 190 Vp in 8 steps while the Power (W) can be selected from 1 to 120 W in 120 steps.

Table S2. Data of average output for each setting

| Average (W) | 15 | 30 | 60 | 90 | 120 |
| --- | --- | --- | --- | --- | --- |
| Bipolar 1.0 (50 Vp, 10 W) | 3.3±0.4 | 3±0 | 3.4±0.2 | 3.7±0.1 | 3.3±0.1 |
| Bipolar 2.0 (70 Vp, 20 W) | 5.6±0.4 | 6.3±0.3 | 8.0±0.2 | 8.3±0.2 | 8±0.2 |
| Bipolar 3.0 (125 Vp, 30 W) | 18.8±1.9 | 21.8±1.3 | 12.5±1 | 7.7±0.2 | 6.5±0.2 |
| Bipolar 4.0 (150 Vp, 40 W) | 26.3±1.0 | 23.3±3.0 | 13.8±1.3 | 8.9±0.2 | 6.4±0.1 |
| Bipolar 5.0 (175 Vp, 50 W) | 34.8±0.5 | 19.5±1.3 | 12±1.8 | 7.8±0.6 | 6.0±0.3 |
| VIO300D (190 Vp, 10W) | 8±0 | 8.3±0.5 | 8±0 | 8±0 | 5.5±0.6 |

Table S3. Data of maximum output for each setting

| Max (W) | 15 | 30 | 60 | 90 | 120 |
| --- | --- | --- | --- | --- | --- |
| Bipolar 1.0 (50 Vp, 10 W) | 3.5±0.4 | 3.2±0.1 | 3.7±0.2 | 4.1±0.1 | 3.8±0.2 |
| Bipolar 2.0 (70 Vp, 20 W) | 6±0.4 | 6.9±0.3 | 9.4±0.1 | 9.3±0.2 | 9.2±0.2 |
| Bipolar 3.0 (125 Vp, 30 W) | 21±2.2 | 25.8±1.3 | 24.5±0.6 | 23.8±0.5 | 23.5±1 |
| Bipolar 4.0 (150 Vp, 40 W) | 28±0.8 | 33.5±2.5 | 36±1.8 | 33.5±1.3 | 35.8±3.1 |
| Bipolar 5.0 (175 Vp, 50 W) | 37.3±0.5 | 40.3±2.6 | 45.3±1.7 | 45.3±3.3 | 45.5±2.5 |
| VIO300D (190 Vp, 10W) | 9.8±0.5 | 10±0 | 9.5±0.6 | 9.3±0.5 | 10±0 |

Table S4. Time until output down in each setting

| Output down (s) | 15 | 30 | 60 | 90 | 120 |
| --- | --- | --- | --- | --- | --- |
| Bipolar 1.0 (50 Vp, 10 W) | － | － | － | － | － |
| Bipolar 2.0 (70 Vp, 20 W) | － | － | － | － | － |
| Bipolar 3.0 (125 Vp, 30 W) | － | － | 34±2 | 28.5±0.6 | 33±4.2 |
| Bipolar 4.0 (150 Vp, 40 W) | － | 24.8±2.1 | 25.5±2.5 | 23.3±1.5 | 24.5±1.7 |
| Bipolar 5.0 (175 Vp, 50 W) | － | 14.5±1 | 17±2.4 | 14±0.8 | 14.5±0.6 |
| VIO300D (190 Vp, 10W)* | － | － | － | － | ＋ |

^*^VIO300D was estimated from the data of average and maximum outputs

Table S5. Detailed data on clinical outcomes of 5 patients with intraductal extension of ampullary neoplasms treated with VIO3-ID-RFA

| Case | Age (years)  / Sex | Tumor size, mm | EP pathology  (subtype) | Intraductal extension (mm) | Intraductal pathology | Complementary interventions before ID-RFA | ID-RFA session | Stent  B-duct  / P-duct | Adverse events | Follow-up period (months) | Recurrence |
| --- | --- | --- | --- | --- | --- | --- | --- | --- | --- | --- | --- |
| 1 | 37  / Male | 22 | High-grade adenoma (intestinal type) | Bile duct  15 | High-grade adenoma (intestinal type) | APC | 1 | FCSEMS  / PS | − | 48 | − |
| 2* | 82  / Male | 16 | Carcinoma in adenoma (intestinal type) | Bile duct  15 | High-grade adenoma (intestinal type) | Snare resection  APC | 1 | FCSEMS  / PS | − | 36 | − |
| 3 | 76  / Male | 16 | High-grade adenoma (intestinal type) | Bile duct  10 | Low-grade adenoma (intestinal type) | APC | 1 | SEMS  / PS | B-duct stricture | 24 | − |
| 4 | 51  / Male | 15 | High-grade adenoma (intestinal type) | Bile duct  8 | Low-grade adenoma (intestinal type) | APC | 1 | SEMS  / PS | B-duct stricture | 24 | − |
| 5 | 57  / Male | 15 | High-grade adenoma (intestinal type) | Bile duct  10 | Low-grade adenoma (intestinal type) | APC | 1 | SEMS  / PS | − | 12 | − |

EP, endoscopic papillectomy; ID-RFA, intraductal radiofrequency ablation; APC, algon plasma coagulation; B-duct, bile duct; P-duct, pancreatic duct; FCSEMS, fully-covered self-expandable metallic stent; PS, plastic stent. *Case 2 is included in a previous publication.

VIO3 setting for ID-RFA: bipolar 3.0 (125 Vp, 30 W) for 30 s.
