# Supplementary figures and images for: Verification in an animal study of the appropriate settings for a novel radiofrequency generator in radiofrequency ablation therapy for residual intraductal lesions after endoscopic papillectomy (with video)
Source: Dig Endosc. 2025 Jan 20;37(6):704–11. doi: 10.1111/den.14986 (PMC12162417; doi:10.1111/den.14986)

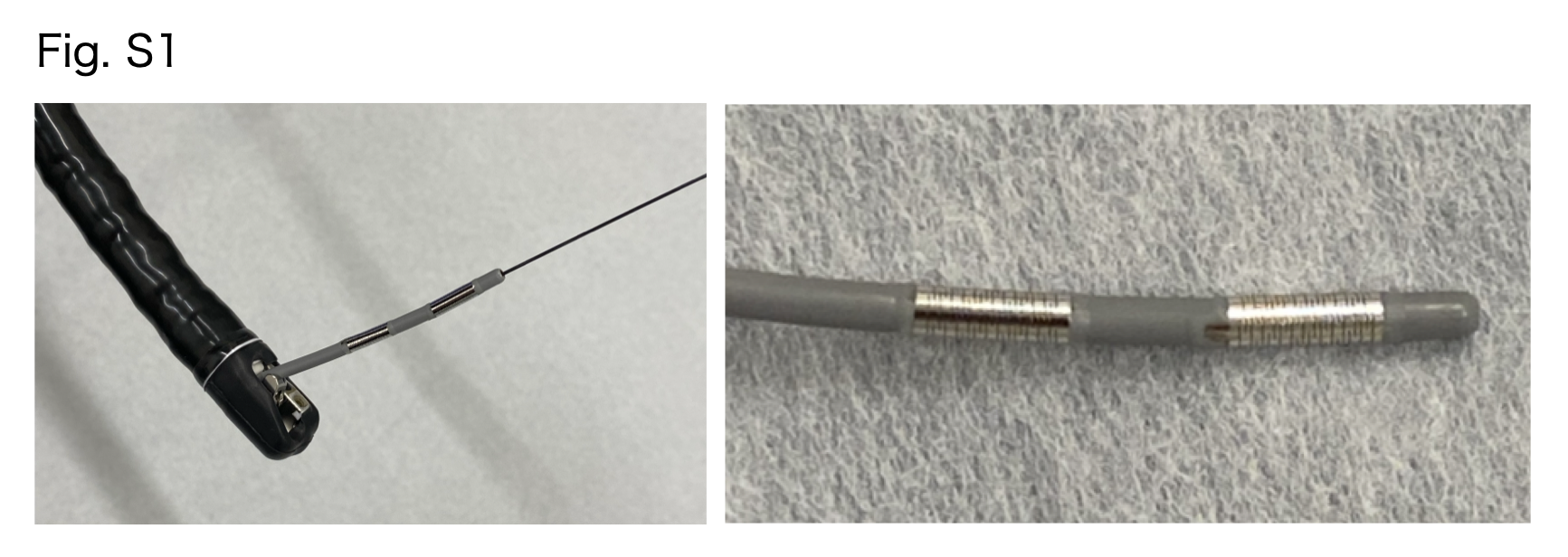

Supplement: Supplementary file 1 — Figure S1 Radiofrequency ablation catheter. Figure S2 Practical in vitro experiment. Figure S3 Ablation effect of 10 W for VIO3 and 10 W for VIO300D (recommended setting). Figure S4 Ablation effects of each voltage with 10 W for VIO300D. Figure S5 Ablation effects of each time for VIO3 at bipolar 3.0 (125 Vp, 30 W). Figure S6 Resected specimen with an ablation time of 60 s stained with hematoxylin and eosin. Figure S7 Observation of temperature change of electrode by thermography. Figure S8 Schema of the spread of the ablation effect during radiofrequency ablation. Figure S9 Ablation effect produced by differences in current density. Figure S10 The demonstration of tips on effective ablation in intraductal radiofrequency ablation using VIO300D with the recommended setting. Table S1 Characteristics of the bipolar settings in each radiofrequency generator. Table S2 Data of average output for each setting. Table S3 Data of maximum output for each setting. Table S4 Time until output down in each setting. Table S5 Detailed data on clinical outcomes of five patients with intraductal extension of ampullary neoplasms treated with VIO3 intraductal radiofrequency ablation. [file DEN-37-704-s002.zip › Fig. S1 R1.tiff]

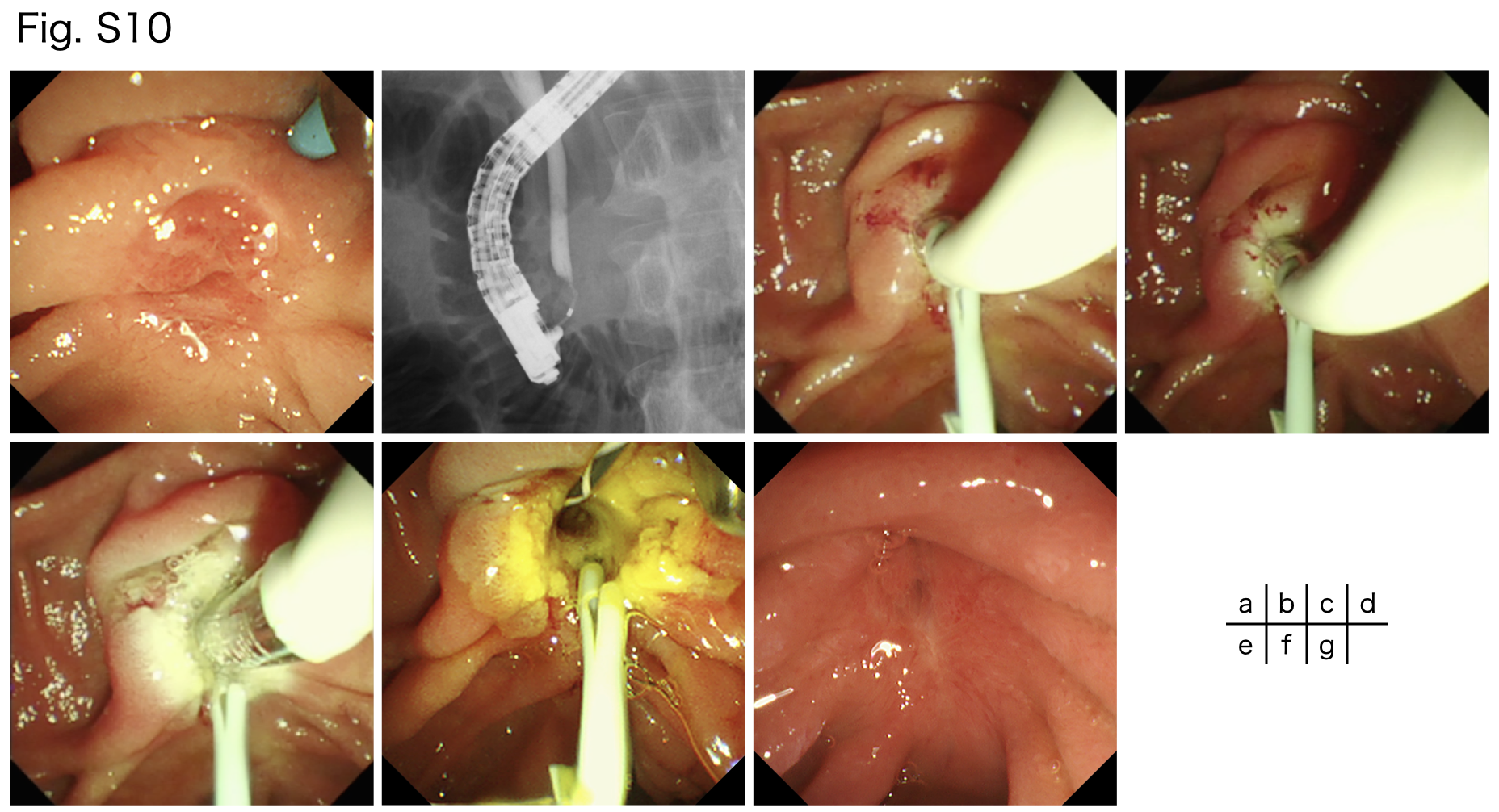

Supplement: Supplementary file 1 — Figure S1 Radiofrequency ablation catheter. Figure S2 Practical in vitro experiment. Figure S3 Ablation effect of 10 W for VIO3 and 10 W for VIO300D (recommended setting). Figure S4 Ablation effects of each voltage with 10 W for VIO300D. Figure S5 Ablation effects of each time for VIO3 at bipolar 3.0 (125 Vp, 30 W). Figure S6 Resected specimen with an ablation time of 60 s stained with hematoxylin and eosin. Figure S7 Observation of temperature change of electrode by thermography. Figure S8 Schema of the spread of the ablation effect during radiofrequency ablation. Figure S9 Ablation effect produced by differences in current density. Figure S10 The demonstration of tips on effective ablation in intraductal radiofrequency ablation using VIO300D with the recommended setting. Table S1 Characteristics of the bipolar settings in each radiofrequency generator. Table S2 Data of average output for each setting. Table S3 Data of maximum output for each setting. Table S4 Time until output down in each setting. Table S5 Detailed data on clinical outcomes of five patients with intraductal extension of ampullary neoplasms treated with VIO3 intraductal radiofrequency ablation. [file DEN-37-704-s002.zip › Fig. S10 R1.tiff]

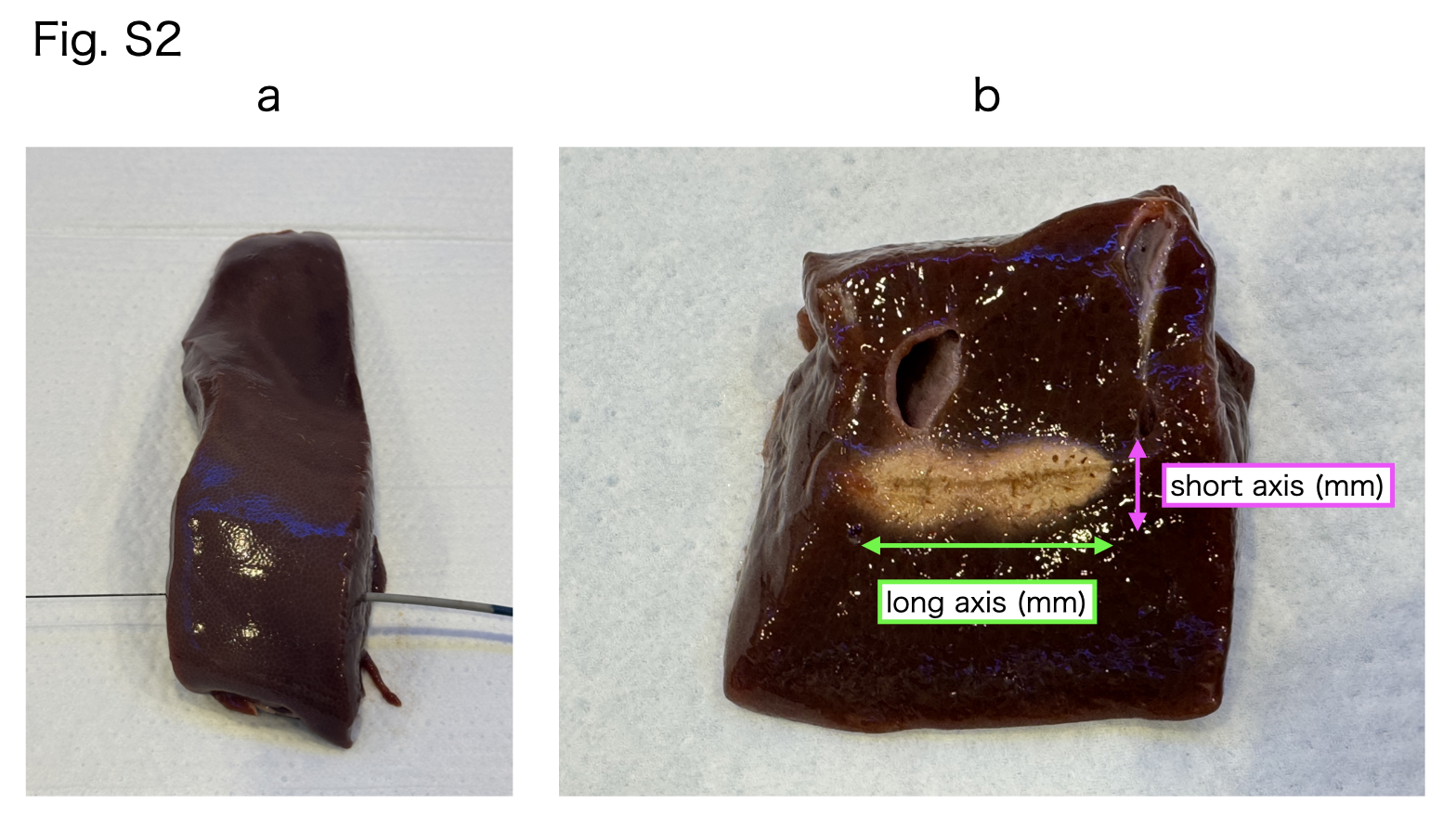

Supplement: Supplementary file 1 — Figure S1 Radiofrequency ablation catheter. Figure S2 Practical in vitro experiment. Figure S3 Ablation effect of 10 W for VIO3 and 10 W for VIO300D (recommended setting). Figure S4 Ablation effects of each voltage with 10 W for VIO300D. Figure S5 Ablation effects of each time for VIO3 at bipolar 3.0 (125 Vp, 30 W). Figure S6 Resected specimen with an ablation time of 60 s stained with hematoxylin and eosin. Figure S7 Observation of temperature change of electrode by thermography. Figure S8 Schema of the spread of the ablation effect during radiofrequency ablation. Figure S9 Ablation effect produced by differences in current density. Figure S10 The demonstration of tips on effective ablation in intraductal radiofrequency ablation using VIO300D with the recommended setting. Table S1 Characteristics of the bipolar settings in each radiofrequency generator. Table S2 Data of average output for each setting. Table S3 Data of maximum output for each setting. Table S4 Time until output down in each setting. Table S5 Detailed data on clinical outcomes of five patients with intraductal extension of ampullary neoplasms treated with VIO3 intraductal radiofrequency ablation. [file DEN-37-704-s002.zip › Fig. S2 R1.tiff]

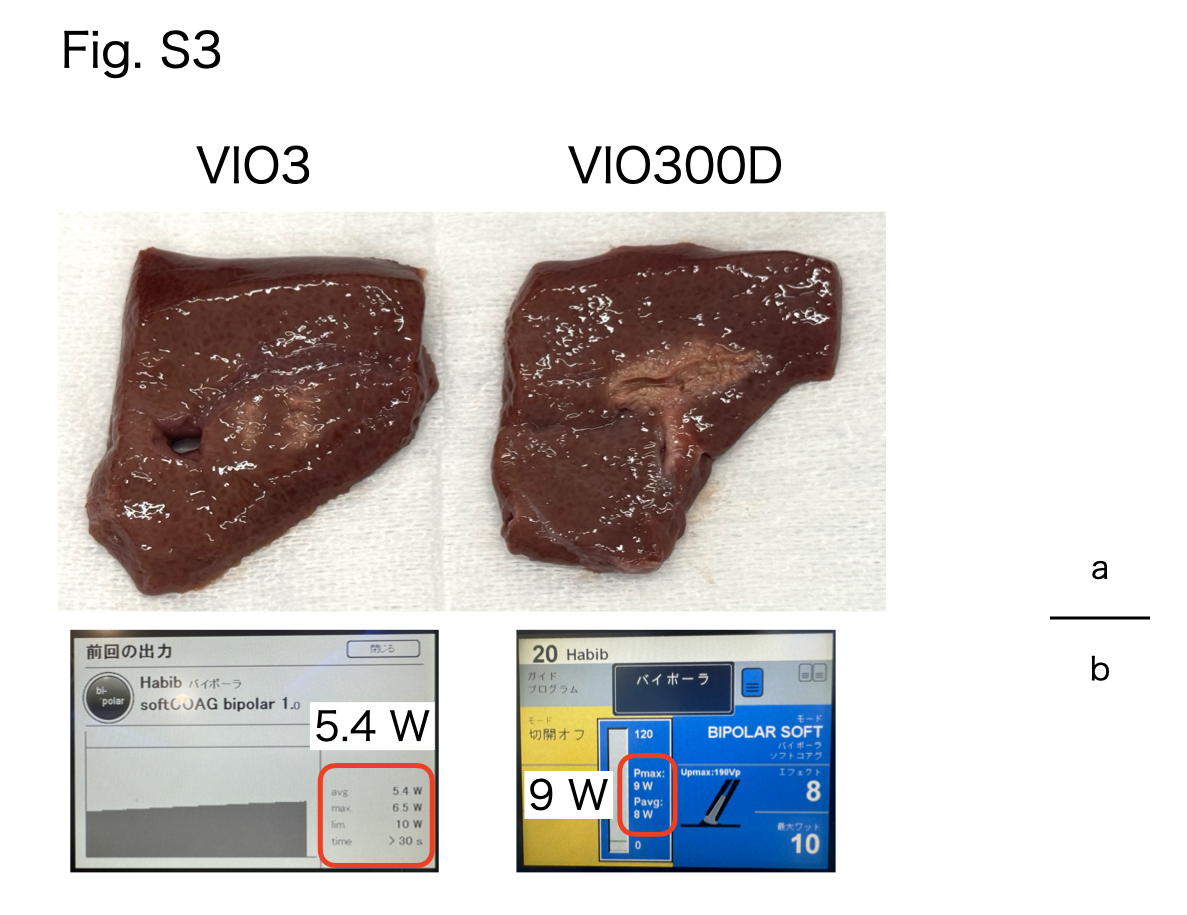

Supplement: Supplementary file 1 — Figure S1 Radiofrequency ablation catheter. Figure S2 Practical in vitro experiment. Figure S3 Ablation effect of 10 W for VIO3 and 10 W for VIO300D (recommended setting). Figure S4 Ablation effects of each voltage with 10 W for VIO300D. Figure S5 Ablation effects of each time for VIO3 at bipolar 3.0 (125 Vp, 30 W). Figure S6 Resected specimen with an ablation time of 60 s stained with hematoxylin and eosin. Figure S7 Observation of temperature change of electrode by thermography. Figure S8 Schema of the spread of the ablation effect during radiofrequency ablation. Figure S9 Ablation effect produced by differences in current density. Figure S10 The demonstration of tips on effective ablation in intraductal radiofrequency ablation using VIO300D with the recommended setting. Table S1 Characteristics of the bipolar settings in each radiofrequency generator. Table S2 Data of average output for each setting. Table S3 Data of maximum output for each setting. Table S4 Time until output down in each setting. Table S5 Detailed data on clinical outcomes of five patients with intraductal extension of ampullary neoplasms treated with VIO3 intraductal radiofrequency ablation. [file DEN-37-704-s002.zip › Fig. S3 R1.tiff]

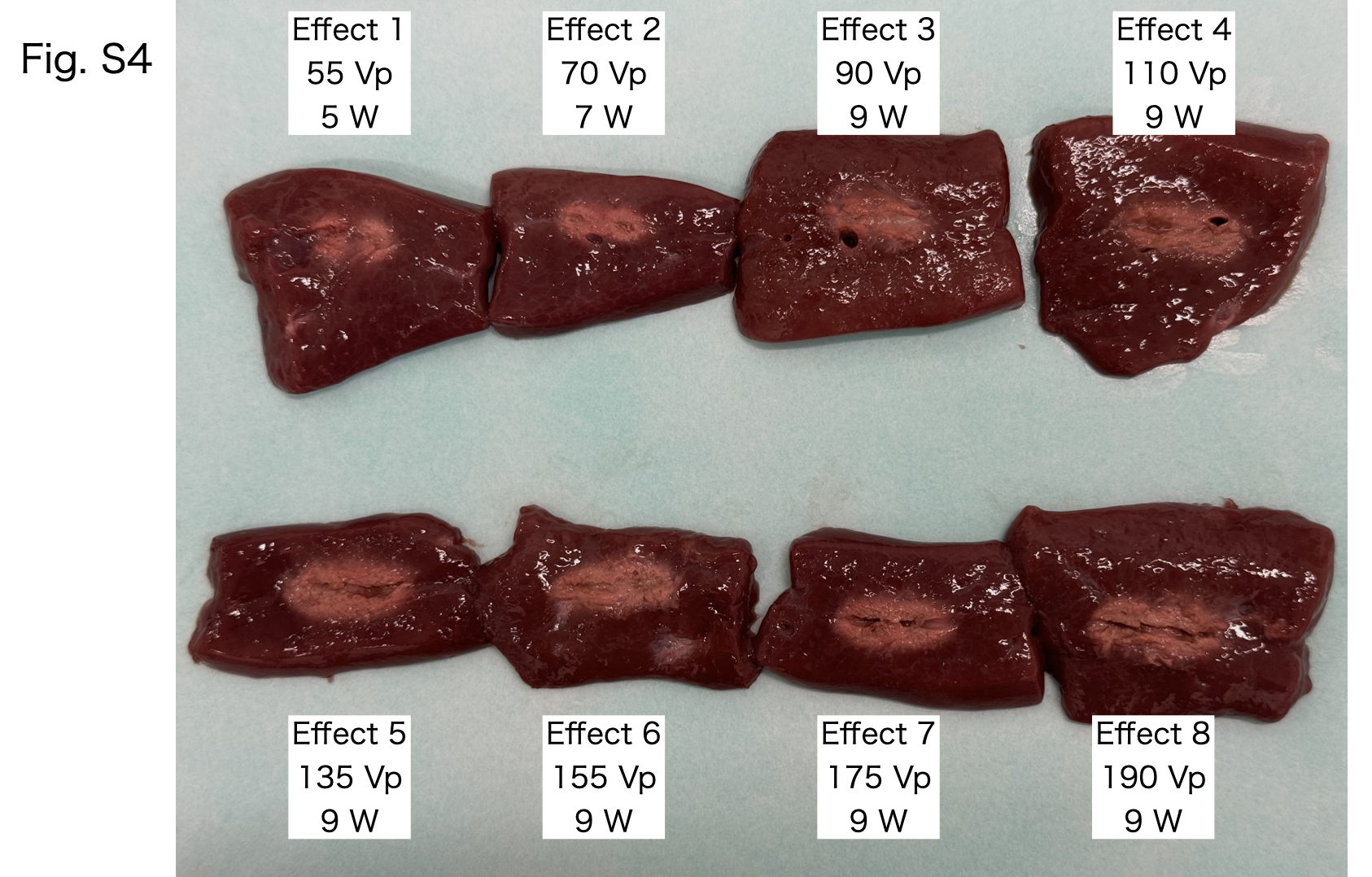

Supplement: Supplementary file 1 — Figure S1 Radiofrequency ablation catheter. Figure S2 Practical in vitro experiment. Figure S3 Ablation effect of 10 W for VIO3 and 10 W for VIO300D (recommended setting). Figure S4 Ablation effects of each voltage with 10 W for VIO300D. Figure S5 Ablation effects of each time for VIO3 at bipolar 3.0 (125 Vp, 30 W). Figure S6 Resected specimen with an ablation time of 60 s stained with hematoxylin and eosin. Figure S7 Observation of temperature change of electrode by thermography. Figure S8 Schema of the spread of the ablation effect during radiofrequency ablation. Figure S9 Ablation effect produced by differences in current density. Figure S10 The demonstration of tips on effective ablation in intraductal radiofrequency ablation using VIO300D with the recommended setting. Table S1 Characteristics of the bipolar settings in each radiofrequency generator. Table S2 Data of average output for each setting. Table S3 Data of maximum output for each setting. Table S4 Time until output down in each setting. Table S5 Detailed data on clinical outcomes of five patients with intraductal extension of ampullary neoplasms treated with VIO3 intraductal radiofrequency ablation. [file DEN-37-704-s002.zip › Fig. S4 R1.tiff]

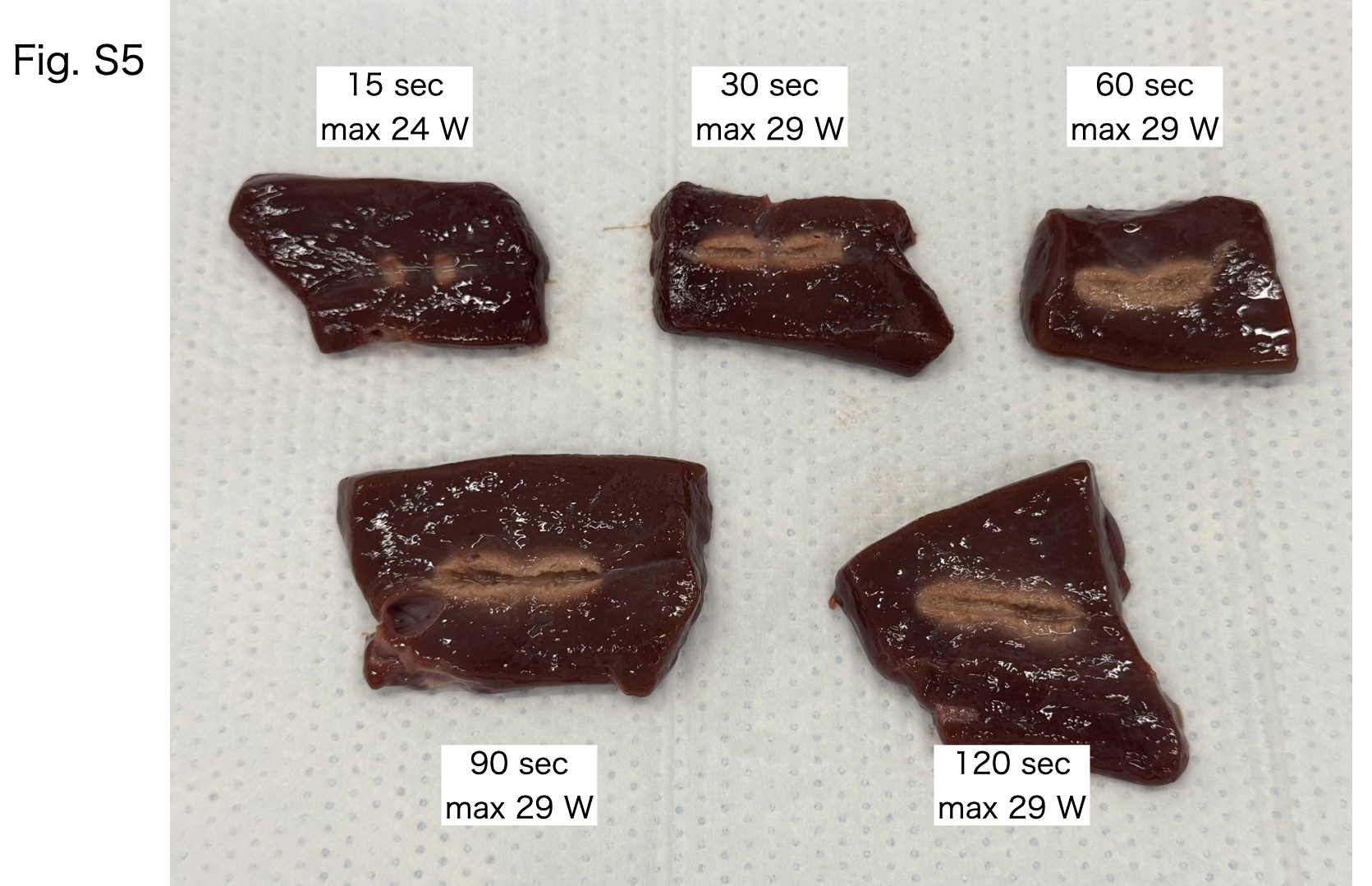

Supplement: Supplementary file 1 — Figure S1 Radiofrequency ablation catheter. Figure S2 Practical in vitro experiment. Figure S3 Ablation effect of 10 W for VIO3 and 10 W for VIO300D (recommended setting). Figure S4 Ablation effects of each voltage with 10 W for VIO300D. Figure S5 Ablation effects of each time for VIO3 at bipolar 3.0 (125 Vp, 30 W). Figure S6 Resected specimen with an ablation time of 60 s stained with hematoxylin and eosin. Figure S7 Observation of temperature change of electrode by thermography. Figure S8 Schema of the spread of the ablation effect during radiofrequency ablation. Figure S9 Ablation effect produced by differences in current density. Figure S10 The demonstration of tips on effective ablation in intraductal radiofrequency ablation using VIO300D with the recommended setting. Table S1 Characteristics of the bipolar settings in each radiofrequency generator. Table S2 Data of average output for each setting. Table S3 Data of maximum output for each setting. Table S4 Time until output down in each setting. Table S5 Detailed data on clinical outcomes of five patients with intraductal extension of ampullary neoplasms treated with VIO3 intraductal radiofrequency ablation. [file DEN-37-704-s002.zip › Fig. S5 R1.tiff]

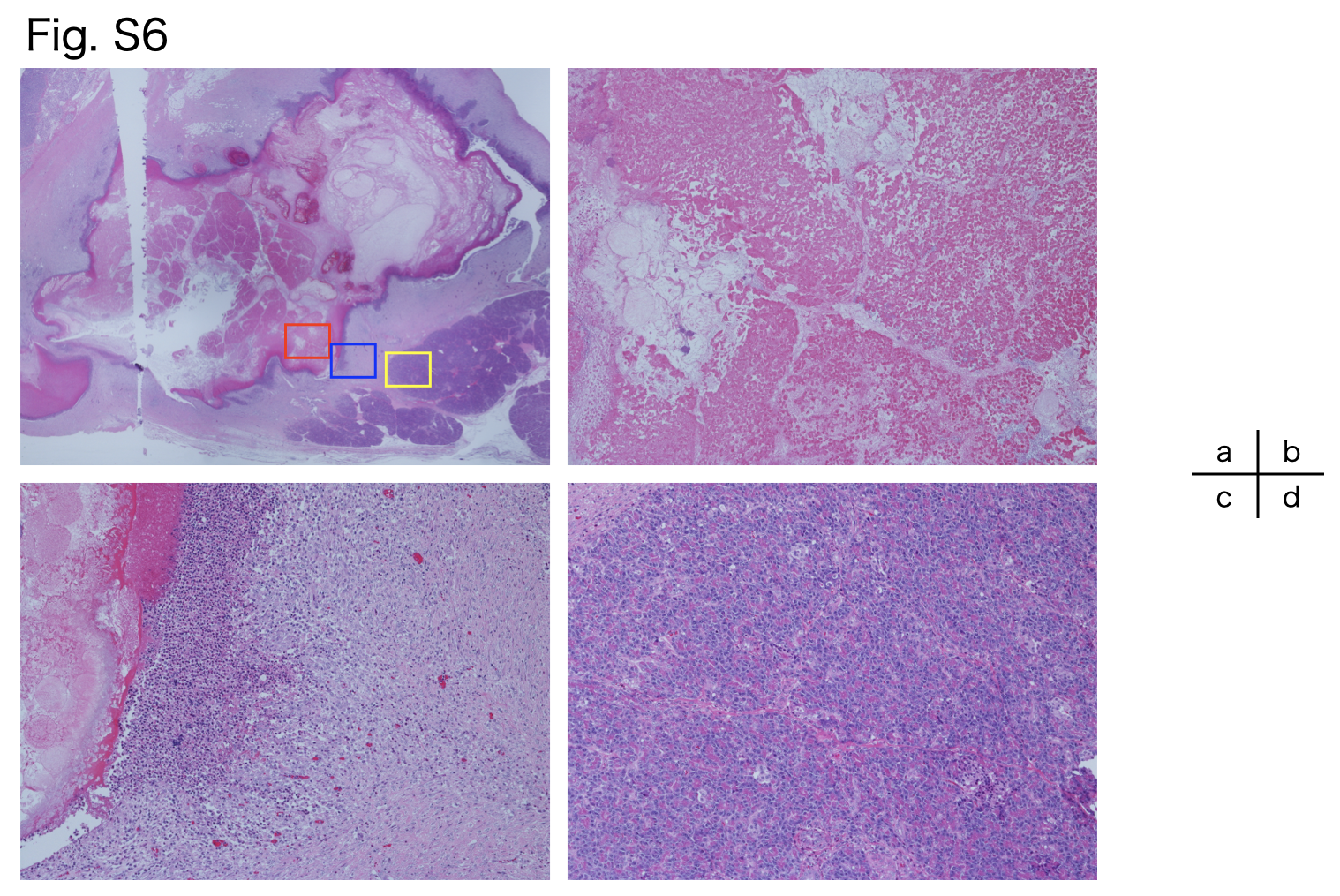

Supplement: Supplementary file 1 — Figure S1 Radiofrequency ablation catheter. Figure S2 Practical in vitro experiment. Figure S3 Ablation effect of 10 W for VIO3 and 10 W for VIO300D (recommended setting). Figure S4 Ablation effects of each voltage with 10 W for VIO300D. Figure S5 Ablation effects of each time for VIO3 at bipolar 3.0 (125 Vp, 30 W). Figure S6 Resected specimen with an ablation time of 60 s stained with hematoxylin and eosin. Figure S7 Observation of temperature change of electrode by thermography. Figure S8 Schema of the spread of the ablation effect during radiofrequency ablation. Figure S9 Ablation effect produced by differences in current density. Figure S10 The demonstration of tips on effective ablation in intraductal radiofrequency ablation using VIO300D with the recommended setting. Table S1 Characteristics of the bipolar settings in each radiofrequency generator. Table S2 Data of average output for each setting. Table S3 Data of maximum output for each setting. Table S4 Time until output down in each setting. Table S5 Detailed data on clinical outcomes of five patients with intraductal extension of ampullary neoplasms treated with VIO3 intraductal radiofrequency ablation. [file DEN-37-704-s002.zip › Fig. S6 R1.tiff]

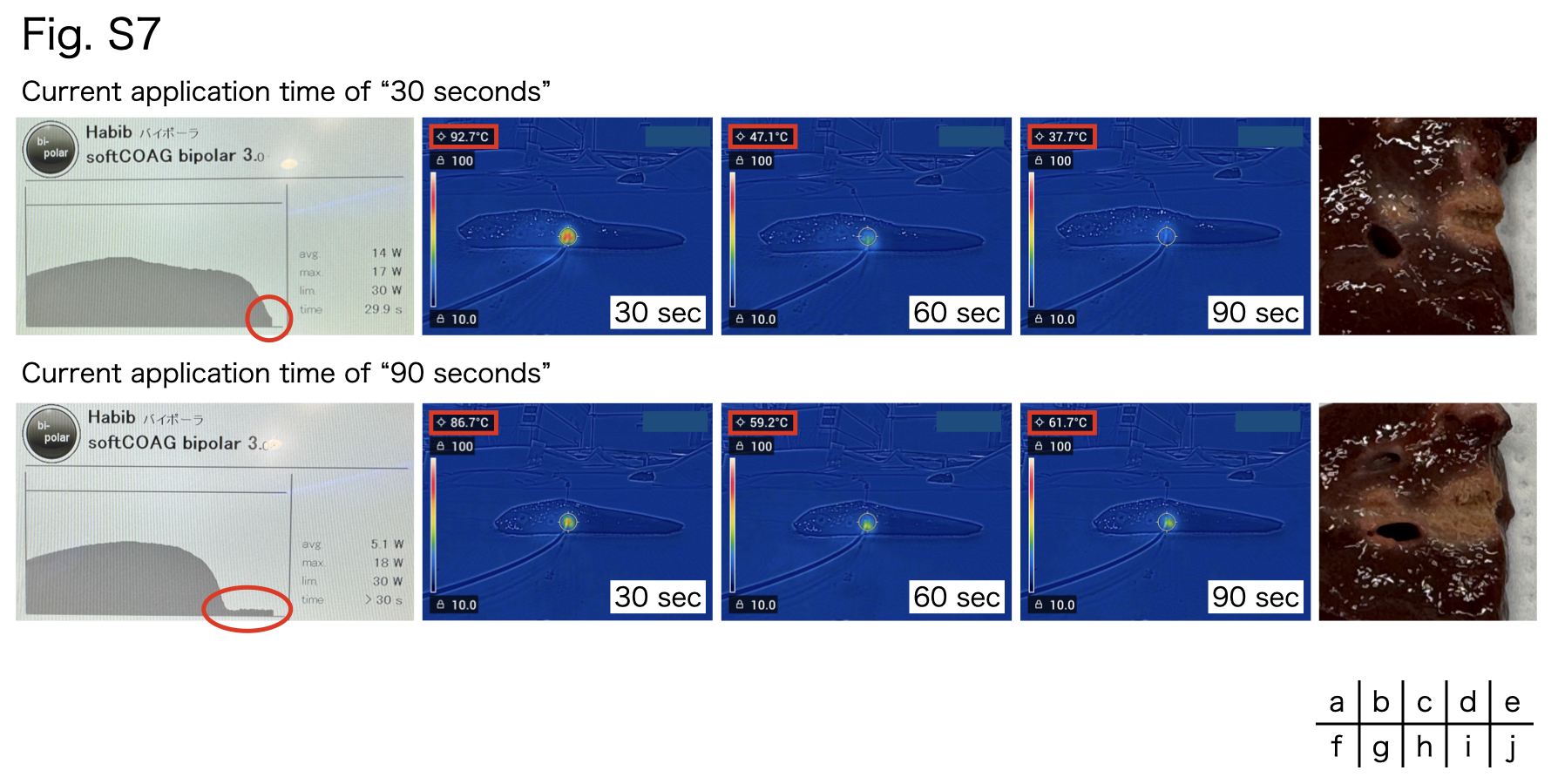

Supplement: Supplementary file 1 — Figure S1 Radiofrequency ablation catheter. Figure S2 Practical in vitro experiment. Figure S3 Ablation effect of 10 W for VIO3 and 10 W for VIO300D (recommended setting). Figure S4 Ablation effects of each voltage with 10 W for VIO300D. Figure S5 Ablation effects of each time for VIO3 at bipolar 3.0 (125 Vp, 30 W). Figure S6 Resected specimen with an ablation time of 60 s stained with hematoxylin and eosin. Figure S7 Observation of temperature change of electrode by thermography. Figure S8 Schema of the spread of the ablation effect during radiofrequency ablation. Figure S9 Ablation effect produced by differences in current density. Figure S10 The demonstration of tips on effective ablation in intraductal radiofrequency ablation using VIO300D with the recommended setting. Table S1 Characteristics of the bipolar settings in each radiofrequency generator. Table S2 Data of average output for each setting. Table S3 Data of maximum output for each setting. Table S4 Time until output down in each setting. Table S5 Detailed data on clinical outcomes of five patients with intraductal extension of ampullary neoplasms treated with VIO3 intraductal radiofrequency ablation. [file DEN-37-704-s002.zip › Fig. S7 R1.tiff]

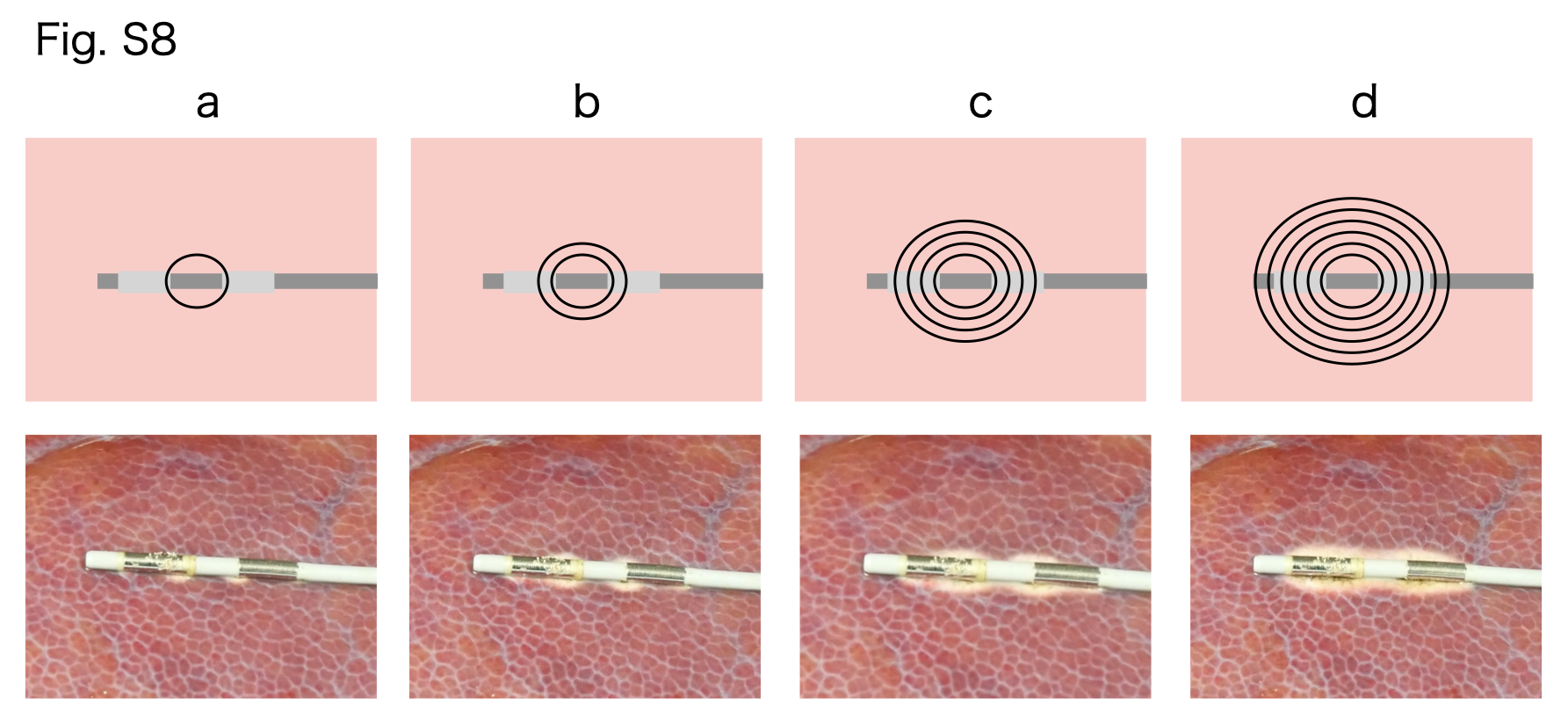

Supplement: Supplementary file 1 — Figure S1 Radiofrequency ablation catheter. Figure S2 Practical in vitro experiment. Figure S3 Ablation effect of 10 W for VIO3 and 10 W for VIO300D (recommended setting). Figure S4 Ablation effects of each voltage with 10 W for VIO300D. Figure S5 Ablation effects of each time for VIO3 at bipolar 3.0 (125 Vp, 30 W). Figure S6 Resected specimen with an ablation time of 60 s stained with hematoxylin and eosin. Figure S7 Observation of temperature change of electrode by thermography. Figure S8 Schema of the spread of the ablation effect during radiofrequency ablation. Figure S9 Ablation effect produced by differences in current density. Figure S10 The demonstration of tips on effective ablation in intraductal radiofrequency ablation using VIO300D with the recommended setting. Table S1 Characteristics of the bipolar settings in each radiofrequency generator. Table S2 Data of average output for each setting. Table S3 Data of maximum output for each setting. Table S4 Time until output down in each setting. Table S5 Detailed data on clinical outcomes of five patients with intraductal extension of ampullary neoplasms treated with VIO3 intraductal radiofrequency ablation. [file DEN-37-704-s002.zip › Fig. S8 R1.tiff]

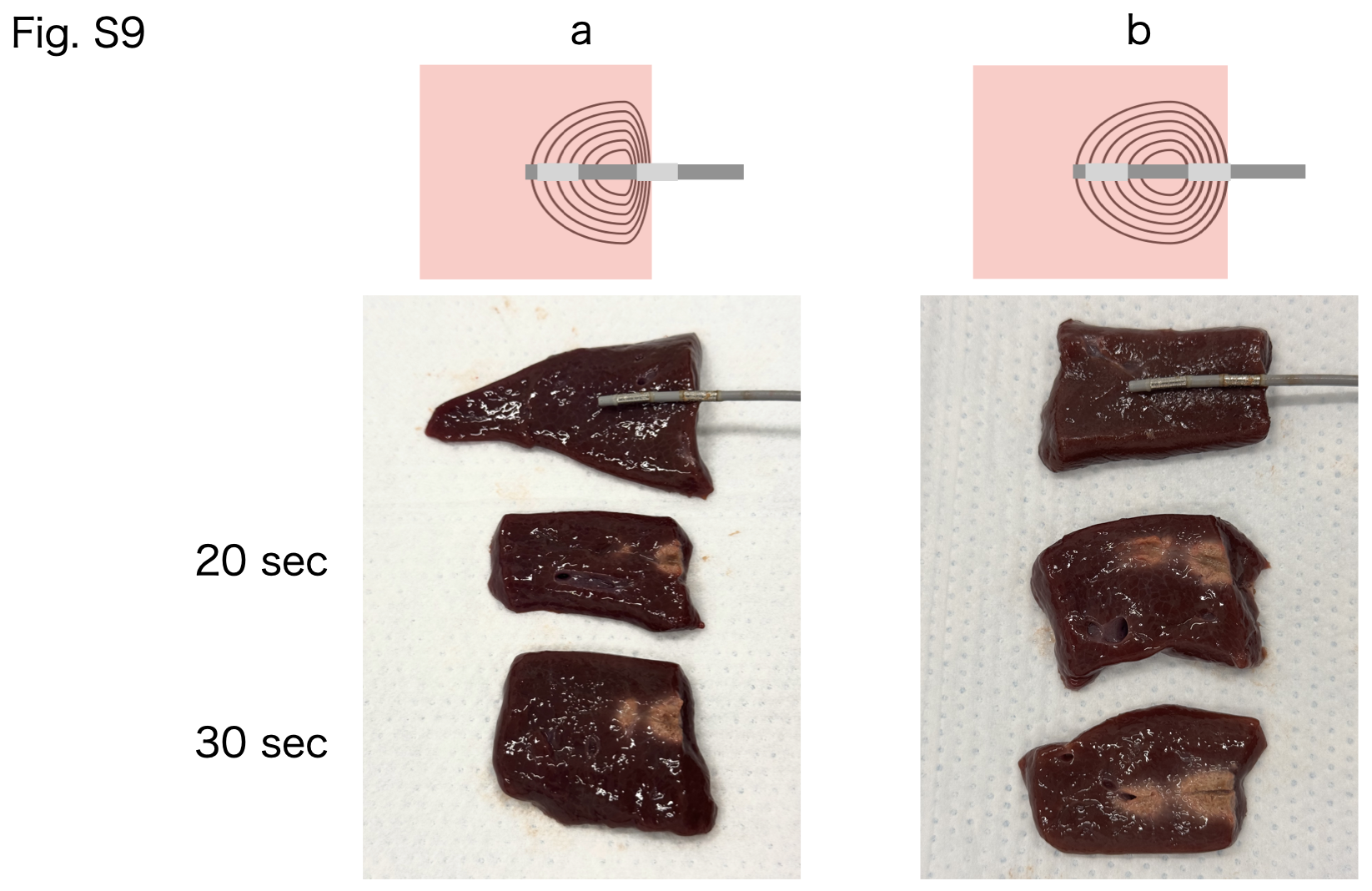

Supplement: Supplementary file 1 — Figure S1 Radiofrequency ablation catheter. Figure S2 Practical in vitro experiment. Figure S3 Ablation effect of 10 W for VIO3 and 10 W for VIO300D (recommended setting). Figure S4 Ablation effects of each voltage with 10 W for VIO300D. Figure S5 Ablation effects of each time for VIO3 at bipolar 3.0 (125 Vp, 30 W). Figure S6 Resected specimen with an ablation time of 60 s stained with hematoxylin and eosin. Figure S7 Observation of temperature change of electrode by thermography. Figure S8 Schema of the spread of the ablation effect during radiofrequency ablation. Figure S9 Ablation effect produced by differences in current density. Figure S10 The demonstration of tips on effective ablation in intraductal radiofrequency ablation using VIO300D with the recommended setting. Table S1 Characteristics of the bipolar settings in each radiofrequency generator. Table S2 Data of average output for each setting. Table S3 Data of maximum output for each setting. Table S4 Time until output down in each setting. Table S5 Detailed data on clinical outcomes of five patients with intraductal extension of ampullary neoplasms treated with VIO3 intraductal radiofrequency ablation. [file DEN-37-704-s002.zip › Fig. S9 R1.tiff]
